# Field boundary delineation with seasonal sentinel 2 imagery using Segment Anything Model (SAM)

**DOI:** 10.1016/j.mex.2026.103794

**Published:** 2026-01-09

**Authors:** Thuan Ha, Kwabena Abrefa Nketia, Hansanee Fernando, Sarah van Steenbergen, Shawn Neudorf, Steve J. Shirtliffe

**Affiliations:** Department of Plant Sciences, College of Agriculture and Bioresources, University of Saskatchewan, Saskatoon, SK S7N 5A8, Canada

**Keywords:** Agriculture, Farm border, Remote sensing, Segment anything model (SAM)

## Abstract

Accurate field boundary delineation is critical for accurate modelling on crop yields and for precision agriculture (PA), enabling site-specific management to optimize resource use and crop productivity. Traditional boundary mapping methods, such as manual digitization and semi-automated extraction from farm machinery, are labor-intensive and challenging to apply at large scales. Advances in high-resolution land cover data and satellite imagery offer scalable solutions for automated field boundary extraction. In this study, we propose a fully automated workflow that integrates a pre-trained foundation model, the Segment Anything Model - SAM [1] with time-series Sentinel-2 imagery. Seasonal composites of Red, Green, and Blue bands were generated at different phenological stages to support segmentation. The method was applied across over 32 million hectares (79 million acres) of cultivated land in the Canadian Prairies, achieving an intersection-over-union (IoU) accuracy of 0.86 compared to manual segmentation. The workflow consists of four main steps: (1) setting the python working environment, (2) seasonal image acquisition and preprocessing using Google Earth Engine via Python API; (3) field boundary segmentation using SAM; and (4) post-processing and feature cleaning using ArcGIS Pro. This approach demonstrates a scalable, efficient solution for large-scale field boundary mapping to support PA applications.

• Integrates a foundation segmentation model (SAM) with Sentinel-2 seasonal imagery
• Demonstrates high-accuracy, large-scale automated field boundary delineation
• Provides a reproducible workflow adaptable to other regions and datasets

## Graphical abstract

**Figure:**
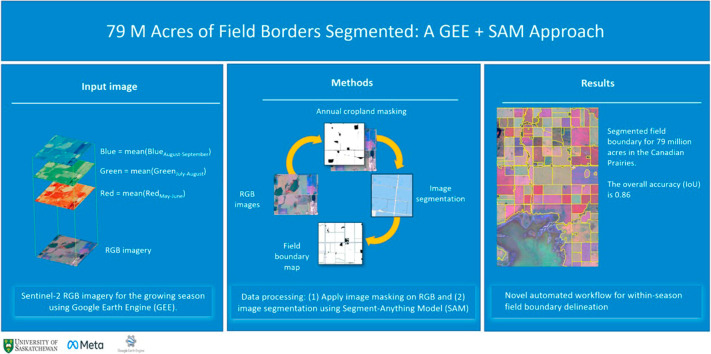

## Specifications table

**Table:** 

**Subject area**	Agricultural and Biological Sciences
**More specific subject area**	Field boundary mapping using Sentinel 2 imagery for geospatial statistics and precision agriculture
**Name of your method**	Cropland field boundary mapping using SAM model with Sentinel 2 time-series imagery.
**Name and reference of the original method**	Reference: Segment Anything Model (SAM). Object detection using deep learning from ArcPy.
**Resource availability**	Software: ArcGIS Pro version 2.9.0 (Esri Inc. 2021); Python 3.7.11 Hardware: Workstation 256 GB RAM, 140 GM GPU, 16-cores processor, internet 267 Mbps downloading and 155 Mbps uploading.

## Background

Accurate delineation of field boundaries is a critical requirement for various agricultural applications, including land information systems, crop monitoring, and precision agriculture. In the Canadian Prairie Provinces, which represent the largest agricultural production region in Canada, no comprehensive and automated field boundary dataset currently exists. Field boundaries are typically obtained through manual digitization, field surveys, or collected indirectly from farm machinery such as combine harvesters, all of which are time-consuming, labor-intensive, and limited in both spatial and temporal coverage. This lack of standardized, scalable boundary data poses challenges for both operational monitoring and research activities that rely on parcel-level analysis.

Recent advances in image segmentation have introduced new tools for automating field boundary extraction. In particular, Meta AI Research has released the SAM, a foundation model that represents a major advancement in artificial intelligence for computer vision tasks [2]. SAM is pre-trained on a massive dataset containing over 1 billion segmentation masks, covering a wide range of object types and imaging conditions. This extensive pre-training enables SAM to perform general-purpose image segmentation in a zero-shot framework, eliminating the need for task-specific fine-tuning or labeled training data for new applications. SAM generates segmentations based on user-provided prompts, which may include points, bounding boxes, or automatically sampled features. Its architecture incorporates transformer-based encoders and promptable mask decoders [3,4], allowing generalization across diverse domains, including natural images, biomedical data, and remote sensing imagery [5].

Although SAM demonstrates strong generalization, its performance on remote sensing imagery may decline due to differences in spectral, radiometric, and textural properties compared to natural images used during pre-training [2,6,7]. To mitigate this, pre-processing methods such as PCA-based dimensionality reduction, high-frequency decomposition, and guided filtering are applied to enhance spatial-spectral features relevant for segmentation [8]. Non-cropland areas—including urban zones, roads, water bodies, forests, and mountains—are masked beforehand to constrain boundary extraction to agricultural land [9,10]. Agricultural masks are created using ancillary datasets such as land cover maps, administrative boundaries, and elevation models. These steps improve alignment between remote sensing inputs and SAM’s internal representations, resulting in more accurate segmentation outcomes [8,11,12]. Prior studies have also demonstrated the feasibility of using high-resolution satellite imagery, including SPOT [10] and Planet [13,14], for agricultural field boundary extraction, further supporting the suitability of remote sensing imagery for this task. While Kirillov et al. [2] reported strong performance in various contexts, SAM can yield suboptimal results in related but distinct applications. As demonstrated in several studies, incorporating user-specific prompts tailored to the application—in this case, field boundary segmentation—can significantly improve SAM’s performance compared to fully automatic segmentation approaches [2,5,8].

This study presents a reproducible and scalable workflow for automated field boundary delineation across the Canadian Prairies, integrating recent advances in remote sensing and deep learning. Sentinel-2 imagery is processed within Google Earth Engine (GEE) to generate seasonal mean composites of Red, Green, and Blue bands, capturing intra-seasonal variability. Non-cropland areas are masked using ancillary datasets to isolate cultivated land, after which the Segment Anything Model (SAM) is applied for segmentation. The final output is a comprehensive vectorized field boundary dataset, accurately delineating ∼ 33 million hectares (79 million acres) of cultivated land with a high accuracy, providing a critical spatial foundation for land information systems, precision agriculture, and geospatial analyses.

## Method details

**Fig. 1:**
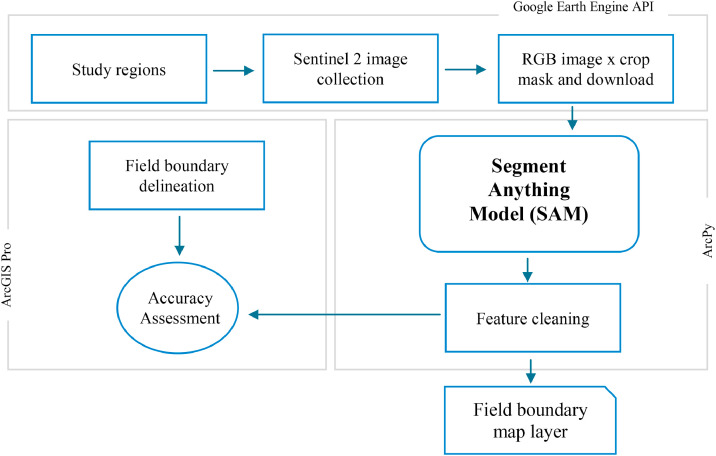
Field boundary detection workflow using segment anything model (SAM).

The field boundary detection workflow consisted of five key steps (Figure 1): (1) creating the Python working environments, (2) acquiring RGB Sentinel-2 imagery, (3) segmenting field boundaries, (4) post-processing the segmented features, and (5) performing accuracy assessment. The workflow was executed across two main working environments: Google Earth Engine (GEE) for image retrieval and preprocessing, and ArcGIS Pro for segmentation and analysis. RGB imagery covering the Region of Interest (ROI) was downloaded via the GEE Python API. SAM was then employed in ArcGIS Pro to delineate field boundaries. Post-processing steps included cleaning and refining the segmented features, which were subsequently evaluated for accuracy. The entire process was implemented using Python-based tools within the configured GEE and ArcGIS Pro environments.

### Step 1: Setting the working environment

To prevent software conflicts, two separate Anaconda Python environments were created: one for downloading Sentinel-2 imagery and another for segmenting field borders. Users must first install the free Anaconda distribution by following the instructions at https://www.anaconda.com. A GEE account is also required and can be created at https://code.earthengine.google.com. All software implementations in this study were carried out on a Windows operating system. A dedicated Anaconda environment was configured using Python 3.10.18 via Anaconda version 4.14.0. Key libraries installed in the environment included: geemap (v0.20.6), GDAL (v3.4.3), GEE API (v0.1.355). The full installation procedure is detailed in the supplementary materials (github link).

• Create the working environment for downloading Sentinel-2 imagery: For example, to create the environment used for downloading Sentinel-2 imagery, the following commands were run in the Anaconda prompt (see Code Block 1).
  (block 1) $$\begin{array}{c} \gg conda\;env\;create-f\;environment.yml\\ \gg conda\;activate\;rgb-environment \end{array}$$
• **Create the working environment for image segmentation**: For the field-boundary segmentation workflow, we used a cloned version of the ArcGIS Pro (v3.3) deep-learning environment. The clone was configured with the Esri Deep Learning Frameworks (per https://github.com/Esri/deep-learning-frameworks). To reproduce this setup, open the ArcGIS Pro Python Command Prompt (Start → ArcGIS → Python Command Prompt) and clone the default arcgispro-py3 environment; the resulting environment includes arcpy and can be customized with the frameworks required for SAM model execution (see Code Block 2).
  (block 2) $$\begin{array}{c} \gg conda\;create\;--field-boundary-seg\;--clone\;arcgispro-py3\\ \gg activate\;field-boundary-seg \end{array}$$

### Step 2: Seasonal image acquisition

To capture seasonal variability in field boundaries, multi-year RGB composites were created using Sentinel-2 imagery from 2021 to 2024. A tiling system based on census subdivision polygons for croplands in the Canadian prairies [15] was applied to parallelize image downloads and structure processing units. Sentinel-2 time-series imagery (May–September) was retrieved using GEE and Python APIs within an Anaconda environment. Preprocessing steps included cloud filtering (retaining scenes with <50% cloud cover), tile-based clipping, temporal aggregation, and masking. RGB bands were generated through median compositing with seasonally adjusted date windows: Red (May–July), Green (July–August), and Blue (August–September), producing annual composites for each tile (Figure 1; Code Block 3). To exclude non-cropland areas, the ESA WorldCover 10 m dataset, AAFC crop inventory map, and road map (Road Network File) from Statistics Canada were incorporated [[16], [17], [18]].

Download process was executed in JupyterLab by navigating to the folder 2_RGB_download and opening the notebook 1a_rgb_download.ipynb. Before running the script, users specified the region of interest (province), the target years, and the output folder path for storing the downloaded imagery. Once configured, the notebook was executed to initiate image downloads. Following download, the images were mosaicked on an annual basis and subsequently masked to remove any remaining non-cropland areas using Otsu thresholding. Additionally, this process produced a shapefile delineating the boundaries of cropland areas. The final mosaics served as input layers for the subsequent image segmentation stage.

(code block 3) $$\begin{array}{c} \gg s2_{jun}=\;s2_{collection}.filter\left(ee.Filter.calendarRange\left(5,\;7,month^{\prime }\right)\right)\;.median\left(\right).select\left(\left[{}^{\prime }B4^{\prime }\right]\right)\\ \gg s2_{jul}=\;s2_{collection}.filter\left(ee.Filter.calendarRange\left(7,\;8,month^{\prime }\right)\right)\;.median\left(\right).select\left(\left[{}^{\prime }B3^{\prime }\right]\right)\\ \gg s2_{aug}=\;s2_{collection}.filter\left(ee.Filter.calendarRange\left(8,\;9,month^{\prime }\right)\right).median\left(\right).select\left(\left[{}^{\prime }B2^{\prime }\right]\right)\\ \gg s2_{img}=\;ee.Image\left(s2_{jun}.addBands\left(s2_{jul}\right).addBands\left(s2_{aug}\right)\right).clip\left(tile_{sub}.geometry\left(\right)\right).3toUint8\left(\right) \end{array}$$

*Where: tile_sub_ is the sub-tile of each major tiling system, s2_collection is the Sentinel-2 image collection for a respective year under segmentation. B4, B3 and B2 are Red, Green and Blue bands of Sentinel-2 imagery respectively.*

### Step 3: Field boundary segmentation using SAM

The SAM is designed to segment clusters of related pixel values within an image. In its baseline design, SAM can segment individual pixels, pixel groups, or bounding boxes. In our study, field boundaries were extracted from Sentinel-2 RGB composites using the Segment Anything Model (SAM) as an automatic proposal generator. Sentinel-2 RGB is free and globally available, and its 10 m resolution supports rapid, large-area processing while preserving sufficient boundary detail. Leveraging SAM’s zero-shot generalization, we avoid task-specific training and labels. For each image tile, SAM generates multiple candidate masks; we retain only stable, high-confidence outputs. The surviving masks are slightly cleaned to yield field polygons. These polygons enable field-scale zonal statistics across the Canadian Prairies. Our use of SAM provides scalable boundary delineation that replaces manual digitization and supports downstream applications—such as zonal statistics—without additional manual digitization.

Image segmentation was conducted within an Anaconda Python environment using the pre-trained SAM model (SAM, 2023). The segmentation workflow was implemented through the 3a_arcpy_rgb_segmentation.ipynb notebook located in the 3_Segmentation directory. Prior to execution, file paths were updated to reference the pre-trained model file (SAM.dlpk) and to define the output directory for storing the resulting shapefiles. The segmentation procedure can be applied either to individual yearly composites or to a multi-year aggregated composite, depending on user-defined settings.

(code block 4) ≫arcpy.ia.DetectObjectsUsingDeepLearning(inRaster,outFeature,sam,parameters)

where: 'inRaster' is the RGB 8-bit image in GeoTIFF format (*.tif), 'outFeature' is the output feature class in shape format (*.shp), 'sam' is the pre-trained model in *.dlpk format, and 'parameters' contains a list of user specified parameters.

Imagery from the four-year period was averaged into a single RGB composite before segmentation. Following code block 4, the object detection function was applied to the input image and the parameter’s name and value were set as in Table 1.

**Table 1:** Parameters used in SAM for field boundary detection.

Name	Value	Explanation
Padding	256	Pixel number added to the image edges
Batch-size	4	Sample number
Box_nms_thresh,	0.7	Threshold for keeping overlapping features
Points_per_batch	64	Sample number per batch
Stability_score_thres	0.95	The level of anomalies
Min_mask_region_area	0	The minimum area of the output features
Run_nms	“NMS”	Keep all output features
Confidence_score_field	“Confidence”	Name of the field in the feature class
Class_value_field	“Class”	Name of the field in the feature class

### Step 4: Post-processing and feature cleaning

**Fig. 2:**
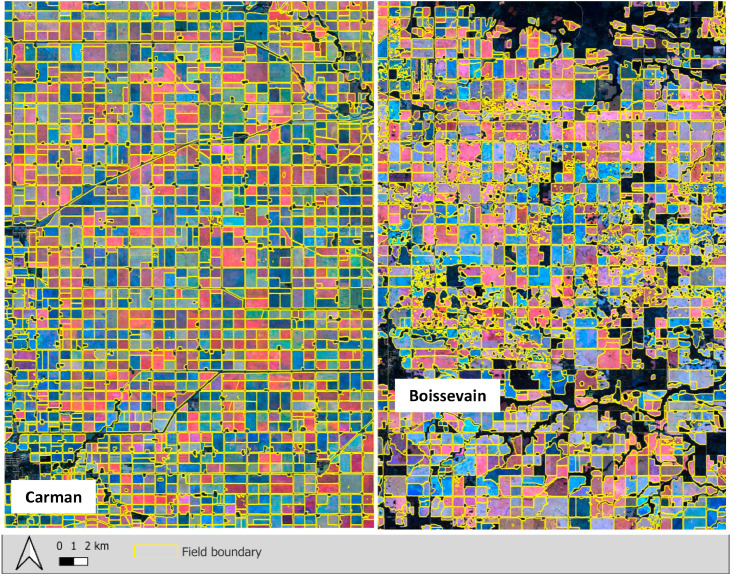
Example of the field boundary output layer (yellow lines) overlaid on a seasonal Sentinel-2 RGB composite.

Feature cleaning consisted of two sequential steps: (1) filtering features based on area, overlap count, and shape compactness, and (2) refining feature boundaries to better align with cropland extents. In the first stage, multiple-year inputs were intersected to align coincident edges and resolve topology; multipart features were exploded; geodesic area computed; and polygons < 2 ha removed. Overlaps were counted to flag ambiguous/shared boundaries, an inward (-20m) buffer trimmed spikes and narrow artifacts, the polygon area was recomputed, and a second ≥ 2 ha filter retained stable footprints. Finally, scale was restored with an outward (+20m) buffer and the cleaned shapefile saved—yielding topologically consistent, size-screened field polygons for downstream analysis. All these task was compiled in 4a_arcpy_clean_shp.ipynb. In the second stage, field-boundary polygons were cross-referenced with the AAFC crop mask, and a zonal mean of the mask values was computed for each polygon. This mean (interpreted as the proportion of cropland within the polygon) was written to the polygon attributes, and polygons with a mean crop-mask value below 0.4 were removed, retaining only fields with a majority cropland signal (4b_cropmask.ipynb) Figure 2.

## Method validation

To evaluate the performance of the field boundary segmentation, we employed the Intersection over Union (IoU) metric, which quantifies the degree of spatial overlap between predicted and reference field boundaries. IoU provides a standardized and interpretable measure of segmentation accuracy, where higher values indicate better alignment between segmented features and ground truth delineated manually from GEE satellite-based map [13]. Accuracy assessments were conducted at 15 randomly selected locations across the Canadian Prairies, encompassing a total area of approximately 260,57 hectares (643,84 acres). The overall mean IoU was 0.86, indicating strong agreement between the segmented and reference boundaries. Province-level evaluations further revealed consistent performance across diverse agricultural landscapes, with mean IoU scores of 0.85 for Saskatchewan (107,95 ha), 0.89 for Alberta (60,43 ha), and 0.86 for Manitoba (92,19 ha). These results confirm the robustness and generalizability of the boundary extraction approach, with slightly higher segmentation accuracy observed in Alberta.

This study presents a semi-automated workflow for large-scale field boundary segmentation across the Canadian prairies, using multi-year Sentinel-2 imagery and the pre-trained SAM. The methodology consisted of environment setup, multi-year image pre-processing and download, RGB composite generation, segmentation, feature cleaning, and boundary refinement. The workflow successfully produced field boundary maps covering the full cropland area of the Canadian prairies, leveraging open-source data and Python-based processing pipelines. The final output consisted of refined field boundary shapefiles generated from the aggregation of three years (2021–2024) of Sentinel-2 RGB imagery. These boundaries captured over 32 million hectares (79 million acres) of cropland. These results indicate high segmentation performance across diverse agricultural landscapes. The workflow demonstrates that field boundary extraction is achievable using pre-trained foundation models combined with open-access satellite imagery and cloud-based geospatial tools. This approach offers strong potential for global application, enabling continuous monitoring of cropland structure and supporting precision agriculture, land use monitoring, and policy assessment. Future work will focus on building labeled training datasets to fine-tune the segmentation model for single-year or intra-seasonal field boundary mapping, further improving accuracy in complex cropping systems.

## Limitations

None.

## Ethics statements

None.

## CRediT author statement

Thuan Ha: Conceptualization, Methodology, Data processing, Software, Writing- Original draft preparation. Kwabena Abrefa Nketia, Hansanee Fernando, Sarah van Steenbergen, Shawn Neudorf: Software, Validity tests, Data curation, Writing- Reviewing and Editing. Steve J. Shirtliffe: Supervision, Funding acquisition, Writing- Reviewing and Editing.

## Related research article

None.

## Supplementary material *and/or* additional information

https://github.com/thuanhavan/CSA_Field_Boundary_Segmentation.

## Declaration of competing interest

Please **tick** the appropriate statement below (please do not delete either statement) and declare any financial interests/personal relationships which may affect your work in the box below.

The authors declare that they have no known competing financial interests or personal relationships that could have appeared to influence the work reported in this paper.

## Data Availability

Data will be made available on request.
